# Polyoxometalate Ligation of PbS Nanocrystals

**DOI:** 10.1021/acs.inorgchem.5c00293

**Published:** 2025-04-28

**Authors:** Talia Ambar, Aranya Kar, Mark Baranov, Nitai Leffler, Alevtina Neyman, Ira A. Weinstock

**Affiliations:** † Dept. of Chemistry, Ben-Gurion University of the Negev, Beer Sheva 84105, Israel; ‡ Ilse Katz Institute for Nanoscale Science & Technology, Ben-Gurion University of the Negev, Beer Sheva 84105, Israel

## Abstract

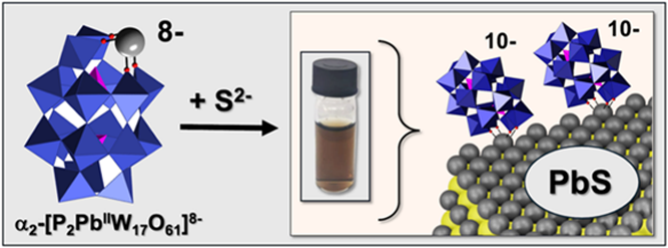

While metal-oxide cluster anions (polyoxometalates, or POMs) stabilize metal nanoparticles (NPs) and metal-oxide nanocrystals (NCs) via established interactions, little is known concerning how POMs might stabilize PbS NCs. Small (ca. 3 nm) Rh and Ir NPs are stabilized “electrosterically”, i.e, via combined electrostatic and steric factors, while larger Au NPs, with less severe curvatures, are protected by electrostatically stabilized monolayers involving the intercalation of countercations between close-packed assemblies of the POM polyanions. By contrast, heteropolytungstates stabilize metal-oxide NCs electrostatically, as observed for charged colloids, but also through direct coordination, wherein monolacunary heteropolytungstate-coordinated metal cations form μ-oxo linkages to metal ions at the NC surface. This raises a general question as to how POMs might stabilize metal-chalcogenide NCs. We now report that room-temperature reaction of the Pb^2+^-substituted monolacunary Wells-Dawson anion, α_2_-[P_2_PbW_17_O_61_]^8–^, with Na_2_S in water provides 4 ± 1 nm PbS NCs. These, in turn, are stabilized by the lacunary ions α_2_-[P_2_W_17_O_61_]^10–^ (**1**) generated via the delivery of Pb^2+^ to sulfide. Unlike POMs on metal NPs, or coordinated via μ-oxo linkages to metal-oxide NCs, the four formally W–O^–^ atoms at the periphery of the defect site of **1** bind to Pb atoms at the (111) surface of the PbS NCs.

## Abstract 2

Polyoxometalate cluster anions (POMs) and their countercations form electrostatically stabilized protecting layers on Au NPs, while POM ligation to metal-oxide NCs via the formation of bridging-oxo linkages gives macroanion-like nanostructures. These findings raise a general question as to how POMs might stabilize metal-chalcogenide NCs. We now show that the monolacunary Wells–Dawson cluster-anion, α_2_-[P_2_W_17_O_61_]^10−^, stabilizes 4 nm PbS NCs in water by out-of-pocket coordination to Pb atoms at their (111) surfaces.

## Introduction

Lead sulfide (PbS) nanocrystals (NCs), prototypical visible-light semiconductors, have recently been utilized in high-efficiency quantum-dot solar cells.^1−6^ One reason for this is that PbS quantum dots feature tunable, particle-size-controlled bandgap energies that provide options for harvesting a compellingly large portion of the solar spectrum, from the visible to near-IR region (700–2500 nm).^5,7^ To stabilize PbS and other metal-chalcogenide NCs, a variety of capping ligands have been used.^3,5,6,8,9^ Most of these are organic, such as 1,2-ethanedithiol (EDT), 1,2-benzenedithiol (1,2-BDT), oleic acid, and 3-mercaptopropionic acid (MPA), although some inorganic ones, such as halides and Sn_2_S_6_^4–^,^3^ have also been reported.^8,9^ Notably, the choice of ligand has considerable influence on the electronic properties of colloidal quantum-dot films and on their performance in optoelectronic devices.^3,5,8,9^

In this context, POMs have yet to be explored as ligands for the controlled synthesis and stabilization of PbS NCs. Moreover, considering the structural motifs by which POM ligands stabilize and solubilize metal(0) NPs^10−13^ and metal-oxide NCs, a fundamental question arises concerning how POM cluster anions might stabilize PbS NCs. A similar question is raised by the reported use of α-[AlW_11_O_39_]^9–^ cluster anions to stabilize CdS NCs.^14^

Relatively small (ca. 3 nm) M(0) NPs of Rh and Ir are stabilized by a combination of electrostatic and steric phenomena,^15^ while larger M(0) NPs, with less severe curvatures, allow for the formation of electrostatically stabilized monolayers of heteropolytungstate cluster-anions and intercalated countercations;^16,17^ closely related hexaniobate anions form coordination-polymer shells^18^ by directly binding to alkali-metal countercations. Notably, POM-monolayer protecting layers can be quite robust, a situation fundamentally different from the ionic strength dependent electrostatic stabilization associated with suspensions of charged colloidal particles.^19^ Finally, heteropolytungsates stabilize metal-oxide NCs^20−22^ in an entirely different fashion. In particular, monovacant Keggin-anion complexed metal cations form μ-oxo (or μ-hydroxo) linkages to metal ions at the NC surface.^23−28^

**Figure 1:**
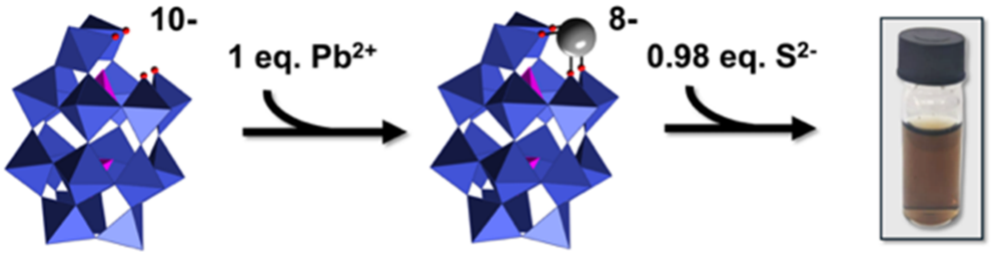
Lacunary Wells–Dawson anion, α_2_-[P_2_W_17_O_61_]^10–^ (**1**), facilitated the synthesis and stabilization of PbS NCs. The synthesis involves formation of a labile out-of-pocket complex, α_2_-[P_2_PbW_17_O_61_]^8–^, followed by incremental addition of Na_2_S. Water and/or OH^–^ ligands on the **1**-complexed Pb^2+^ ion^29^ are not shown.

We now show that the monodefect (lacunary) Wells–Dawson cluster-anion, α_2_-[P_2_W_17_O_61_]^10–^ (**1**, at left in Figure 1) can be used both to prepare water-soluble 4 nm PbS NCs and to subsequently stabilize them via interactions distinctly different from those documented for POM ligands on M(0) NPs or metal-oxide NCs.

## Results and Discussion

### POM Delivery of Pb^2+^ to Sulfide

Synthesis of the POM-complexed PbS NCs was carried out by first adding 1 equiv of Pb(NO_3_)_2_ to an aqueous solution of **1**, followed by the slow addition of 0.98 equiv. Na_2_S with vigorous stirring at room temperature (Figure 1). The slow addition and slightly substoichiometric amount of Na_2_S added were required to ensure the stability of **1** by avoiding increase in the pH to above 7.5.

**Figure 2:**
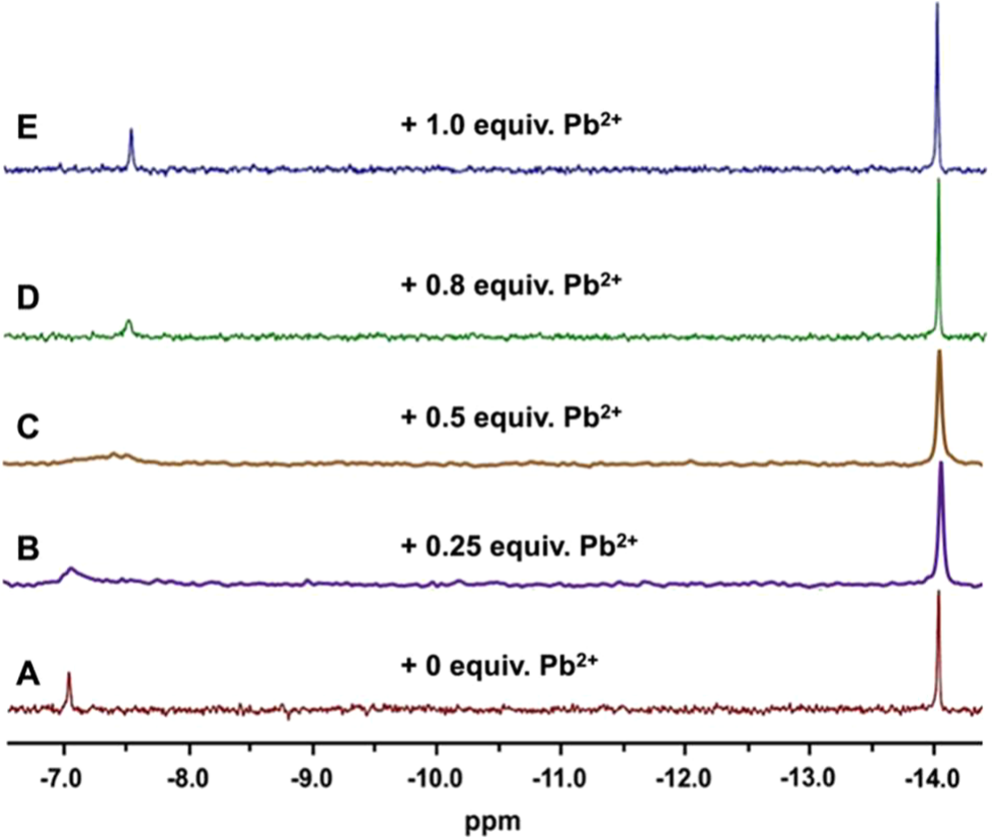
^**31**^P NMR spectra of **1** in the presence of (A) no Pb^2+^, (B) 0.25 equiv of Pb^2+^, (C) 0.5 equiv of Pb^2+^, (D) 0.8 equiv of Pb^2+^ and, (E) one equiv of Pb^2+^. In all the spectra, the signals at −14.0 and ca. −6 to −7 ppm, respectively, arise from P atoms distal and proximal to the monovacant site of **1**. The addition of 0.25 equiv of Pb^2+^ (spectrum B) results in broadening of the downfield signal, while two broad signals are observed when 0.5 equiv of Pb^2+^ is added (spectrum C; see text for more discussion). The broadening decreased upon addition of 0.8 equiv of Pb^2+^ (spectrum D), and addition of one equiv of Pb^2+^ (spectrum E), resulted in a sharp peak corresponding to 1:1 complexation of Pb^2+^ by **1**.

The first step, metalation of **1**, was monitored by ^31^P NMR spectroscopy to gain insight into the strength and nature of the out-of-pocket interaction between monovacant **1** and Pb^2+^ in water. Upon incremental additions of Pb^2+^ (Figure 2), the signal arising from the P atom proximal to the monodefect binding site of **1** shifts upfield and broadens, indicative of exchange between saturated and unsaturated **1**.

Broadening and a small upfield shift is observed when 0.25 equiv of Pb^2+^ are added (spectrum B), while after the addition of 0.5 equiv of Pb^2+^ (spectrum C), two broad signals are observed at ca. −7 and −7.3 ppm. The presence of these two signals may indicate the presence of different complexes, one involving coordination of two anions, **1**, to a single Pb^2+^ atom, and the second to a 1:1 complex. Finally, when one equiv of Pb^2+^ is added (Figure 2, spectrum E), the downfield signal sharpens as the rate of exchange is reduced to a small value due to thermodynamically favorable coordination of Pb^2+^ by the lacunary binding site of single equiv of **1**. At the same time, the lability indicated by the broadening in Figure 2, panels B–D, is expected for complexation of the “soft” Lewis-acid Pb^2+^ ions by the “hard” Lewis-base oxo-donor ligands at the periphery of the defect site of **1**.

Because Pb^2+^ ions are too large to enter into the lacunary site, the Pb^2+^ ions are complexed in an “out-of-pocket” fashion by the four terminal oxide ligands surrounding the defect site of **1**.^29−32^ Given its large, 2.66 Å crystallographic diameter (for the six-coordinate ion), Pb^2+^ can support coordination numbers up to 12, rendering the complexed Pb^2+^ ions^29^ kinetically facile targets for interaction with and transfer to unmetalated POM cluster-anions. Taken together, the favorable, yet labile and sterically accessible, out-of-pocket coordination of Pb^2+^ by **1** (Figure 2), combined with the large size of the Pb^2+^ ions, provide for their controlled delivery to S^2–^. This dematalation of **1** was confirmed by ^31^P NMR spectroscopy (Figure S1).

After in situ generation of **1**-complexed Pb^2+^, the slow addition of 0.98 equiv of Na_2_S gave a brown, optically transparent pH 7–7.5 solution of PbS NCs. In the absence of **1**, reaction of Pb^2+^ with S^2–^ gives insoluble cubic-phase PbS (Figure S2) as a black precipitate with a *K*_sp_ value of 3.0 × 10^–28^. When prepared using **1**, however, a clear solution was obtained, within which **1** was the only heteropolytungstophosphate cluster-anion observed by ^31^P NMR spectroscopy; no other ^31^P signals were observed.

**Figure 3:**
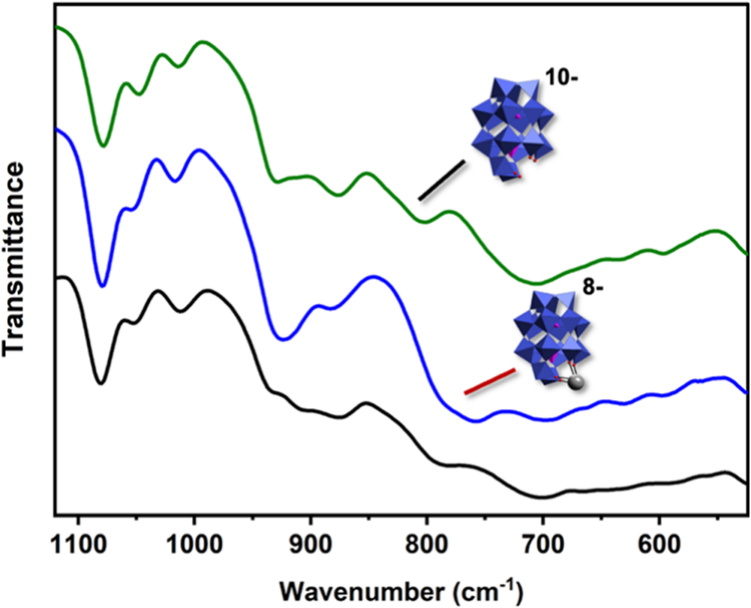
ATR FTIR spectra of **1** (green curve), α_2_-[PbP_2_W_17_O_61_]^8–^ (blue curve), and **1**-complexed PbS NCs (black curve). The representative peaks of the Wells-Dawson anions are observed between 700–1100 cm^–1^.

The product was characterized by methods used in previous work to determine the nature of POM-stabilized M(0) NPs^33−36^ and metal-oxide NCs.^23−28,37^ The main elements, W(VI), Pb(II), and S(II), were observed by X-ray photoelectron spectroscopy (XPS; Figure S3). The synthetic reaction was carried out under conditions of pH and temperature at which **1** and its complexed-Pb derivative are both stable (as confirmed by ^31^P NMR; see Figure 2). Additional confirmation was provided by attenuated total reflectance (ATR) FTIR spectroscopy (Figure 3), obtained after using the addition of EtOH to precipitate most of the nonbound lacunary anions, α_2_-[P_2_W_17_O_61_]^10–^ (**1**), as described below in the Experimental Section.

By measuring the sizes of 95 particles in a wide-area cryo-TEM image of a solution of **1**-stabilized PbS NCs, an average size of 4 ± 1 nm was obtained (Figure S4). As expected for the room-temperature reaction of Pb^2+^ with S^2–^ at ambient pressure,^38^ the NCs are cubic-phase PbS (see Figures S5 and S6 for electron-diffraction and high-resolution TEM data).

**Figure 4:**
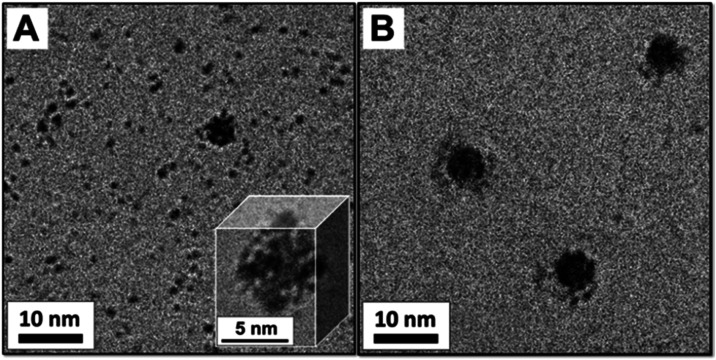
Cryo-TEM images of **1**-complexed PbS NCs. (A) is a solution sampled immediately after synthesis, with excess **1** present in the background; Inset is a slightly enlarged view of a single particle, and (B) is the supernatant solution obtained after selective precipitation of **1** by addition of EtOH, followed by centrifugation to selectively remove **1**.

The presence of compound **1** at the NC surfaces was documented by cryo-TEM imaging (Figure 4). Immediately after synthesis, **1** is observed at the surface of PbS NCs, with excess **1** seen as much smaller objects in the surrounding vitrified-water matrix (Figure 4A and inset). To be certain that the POMs observed at the PbS NC surface were not due to excess **1** randomly observed near or around the NCs, EtOH was used to selectively precipitate most of the excess **1** left from synthesis, after which ca. 1.5 nm sized clusters of **1** were observed by cryo-TEM at NC surfaces (Figure 4B). To our knowledge, these are the first reported images of ligands on a metal-chalcognide NC in its native, vitrified, solution state. Also, in both panels of Figure 4, larger than average PbS NCs were imaged to enhance the contrast of the POM ligands. Notably, POMs at the surfaces of NCs less than ca. 5 to 6 nm in diameter are not typically observed, at least by us, in cryo-TEM images.^23−28,37^ The ζ-potential of −45 eV was consistent with that of **1** at the NC surfaces.

### Ligation of α_2_-[P_2_W_17_O_61_]^10–^ (1) to PbS NCs

The basis for the association of **1** with PbS NC surfaces was then explored. For this, K^+^ salts of four cluster anions were screened for their abilities to stabilize colloidal PbS NCs (Table 1). The cluster anions used were chosen to control for two parameters: (1) the presence of a lacunary binding site and (2) anion charge.

**Table 1:** Effects of Lacunary Binding Sites and Cation Charge on Colloidal-PbS Stability^a^

entry	cluster-anion	charge	presence of lacunary site	colloidal stability
1	α_2_-[P_2_W_17_O_62_]^10–^ (**1**)	10–	yes	stable
2	α-[PW_11_O_39_]^7–^	7–	yes	stable
3	α-[AlW_12_O_40_]^5–^	5–	no	unstable
4	α-[AlV^IV^W_11_O_40_]^7–^	7–	no	unstable

a Stability criteria are described in the Experimental section of the Supporting Information.

As shown in Figure 1, 1 and 0.98 equiv of Na_2_S gave a clear solution (Table 1, entry 1). An analogous result (entry 2) was obtained when the monolacunary anion, α-[PW_11_O_39_]^7–^ was used. PbS was then prepared using the plenary-Keggin ion, α-[AlW_12_O_40_]^5–^, featuring no lacunary site, and stable at pH values up to 7.5.^39^ Use of α-[AlW_12_O_40_]^5–^ gave an unstable suspension of PbS (entry 3). Although this pointed to a role for the lacunary sites of **1** and α-[PW_11_O_39_]^7–^, the 5- charge of α-[AlW_12_O_40_]^5–^ is much smaller than the 10– and 7– charges of **1** and α-[PW_11_O_39_]^7–^ (entries 1 and 2). Notably, small differences in anion charge have significant effects on the abilities of POM anions to stabilize nanoparticles, such as those of gold.^16,35^ To control for anion charge, PbS NCs were prepared using α-[AlV^IV^W_11_O_40_],^7−40^ which features the same negative charge as α-[PW_11_O_39_]^7–^ but lacks a lacunary site. As observed for α-[AlW_12_O_40_]^5–^, α-[AlV^IV^W_11_O_40_]^7–^ gave an unstable suspension of PbS (entry 4). This suggested that the lacunary site of α-[PW_11_O_39_]^7–^, rather than its 7– charge, was responsible for stabilizing the PbS NCs. As such, the screening experiments in Table 1 pointed to a specific stabilizing role for the lacunary sites of **1** and α-[PW_11_O_39_]^7–^.

**Figure 5:**
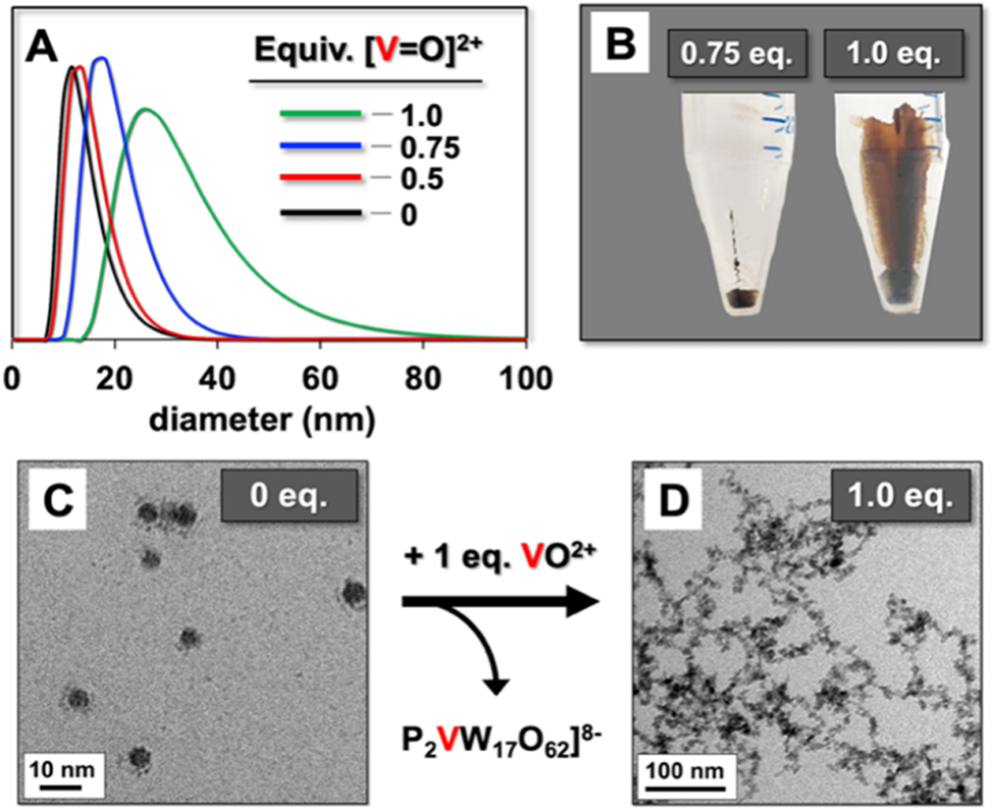
Occupation of the binding site of **1** by the addition of VO^2+^. (A) Volume-percent DLS data after adding VO^2+^ to identical solutions of **1**-stabilized PbS NCs: 0 equiv (black curve), 0.50 equiv (red curve), 0.75 equiv (blue curve), and 1.0 equiv (green curve). (B) Photos of vials showing the amounts of PbS that precipitated after additions of 0.75 and 1.0 equiv of VO^2+^ to identical solutions of **1**-stabilized PbS NCs. (C) Cryo-TEM images of **1**-stabilized PbS NCs with no added VO^2+^. For clarity, the image was obtained after removing excess **1** as discussed in relation to Figure 3B. (D) Cryo-TEM image obtained after adding one equiv of VO^2+^ to a freshly prepared solution of **1**-stabilized PbS NCs.

Definitive evidence that the lacunary site of **1** plays a central role in NC stabilization was obtained by preparing **1**-stabilized PbS NCs, and then incrementally “blocking” the cluster anion’s defect site by in situ complexation with vanadyl ion, [V^IV^=O]^2+^ (Figure 5). Four identical solutions of **1**-stabilized PbS NCs were treated with 0.25, 0.5, 0.75, and 1.0 equiv of the vanadyl ion, VO^2+^, to respectively convert 25, 50, 75 and 100% of the lacunary anions, α_2_-[P_2_W_17_O_61_]^10–^ (**1**) to *pseudo*-plenary anions, α_2_-[P_2_V^IV^W_17_O_62_]^8–^ (Figure 5).

No precipitation was observed upon additions of 0.25 or 0.50 equiv of VO^2+^, and only a slight increase in size (as volume percent) was observed by DLS after addition of 0.50 equiv. (Figure 5A, red curve; intensity-percent DLS data are provided in Figure S7). When 0.75 equiv of VO^2+^ was added, however, considerable aggregation was observed by DLS (blue curve in Figure 5A) and not all of the material could be redissolved after one cycle of salting out and centrifugation (at left in Figure 5B). When 1.0 equiv of VO^2+^ was added, however, extensive aggregation was indicated by DLS (green curve in Figure 4) and all of the material remained insoluble after one cycle of salting out and centrifugation (at right in Figure 5B).

The vanadyl ions were added as the sulfate salt, VOSO_4_, such that an increase in ionic strength could arguably have led to NC instability. This was ruled out by increasing the ionic strength of a solution of **1**-stabilized PbS NCs by addition of NaCl to equal that supplied by one equiv of VOSO_4_. No change was observed by DLS. In addition, clear solutions of POM-stabilized PbS were obtained when prepared using monolacunary α-[PW_11_O_39_]^7–^ (entry 2 in Table 1), which has a smaller charge than the *pseudo*-plenary, vanadyl-substituted anion, α_2_-[P_2_V^IV^W_17_O_62_]^8–^, formed upon addition of VO^2+^. This demonstrated that the instability and precipitation observed in Figure 5A,B were clearly caused by occupation of the lacunary site of **1** by VO^2+^, rather than the attendant decrease in POM-anion charge. Therefore, the lacunary site of **1** plays a critical role in the stabilization and solubilization of the PbS NCs.

This conclusion was supported by cryo-TEM imaging of solutions of **1**-stabilized PbS NCs before and after occupation of the lacunary site of **1**. The addition of 1.0 equiv of VO^2+^ rapidly converted the individual PbS NCs (Figure 5C) into a cross-linked one-dimensional aggregate (Figure 5D). Additional cryo-TEM images, including those for 0.75 equiv of VO^2+^, are provided in Figures S8 and S9.

**Figure 6:**
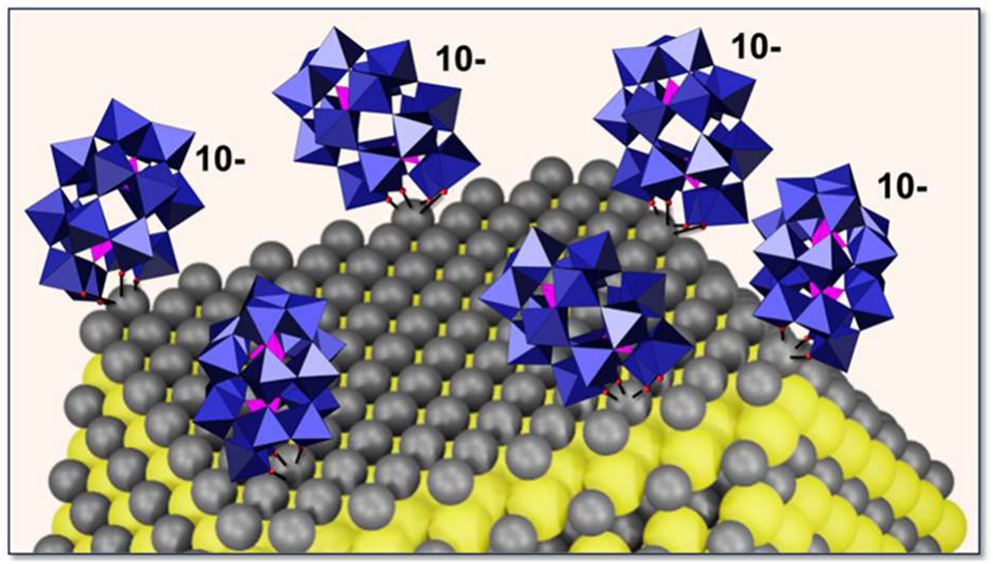
“Out-of-pocket” coordination of the formally W–O^–^ atoms at the periphery of the defect site of α_2_-[P_2_W_17_O_61_]^10–^ (**1**) with 2.66 Å diameter Pb atoms (in gray) at the (111) surface of a PbS NC, relative to which the polyhedral representations of **1** are drawn to scale. Sulfide atoms are in yellow.

The data in Figure 5 show that the lacunary site of **1**, featuring four formally W–O^–^ moieties, plays a central role in stabilizing the PbS NCs. The NCs themselves feature sulfur-exposed (100) surfaces and Pb-terminated (111) faces, at which anionic ligands are known to bind.^41^ This, in combination with a critically important stabilizing role specifically assigned to the presence of a lacunary site, suggests that **1** binds to the relatively large, 2.66 Å diameter, Pb atoms at the (111) surfaces of the NCs (Figure 6).

### Comparison of 1-Stabilized PbS with POM Stabilization of Metal(0) and Metal-Oxide NPs

These findings show that heteropolytungstate cluster-anion stabilization of PbS NCs is entirely different from the structural motifs documented for Au NPs or metal-oxide NCs.

On Au NPs in water, POMs and their countercations generally form electrostatically stabilized monolayers,^16^ although for 3 nm Au NPs in MeCN, highly charged trilacunary γ-[SiW_9_O_34_]^n–^ anions solubilized by tetraoctylammonium cations might interact differently.^12^

Monolacunary heteropolytungstate stabilization of metal-oxide NCs, (M_*x*_O_*y*_)_*z*_,^23−28^ occurs via the in situ pentacoordinate (“in-pocket”) or tetracoordinate (“out-of-pocket”) complexation of **M** atoms, and the formation of bridging-oxo linkages between the POM-complexed **M** atoms and **M** atoms at the NC surface. This is illustrated in eq 1 for NC formation and stabilization by monolacunary α-[PW_11_O_39_]^7**–**^.
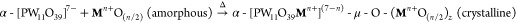
1

For POM stabilization of metal chalcogenides, (M_*x*_S_*y*_)_*z*_, generally, the critical difference is that sulfide linkages to POM-coordinated transition metals, e.g., [PW_11_O_39_**M**^*n*+^]^(7–*n*)^–μ-S^2**–**^, are readily hydrolyzed in water. As such, rather than forming bridging-sulfide linkages analogous to the μ-oxo linkage in eq 1, the defect site of the POM anions bind directly to Pb atoms at the NC surface, as shown in terms of α_2_-[P_2_W_17_O_61_]^10**–**^ (**1**) in eqs 2 and 3, and Figure 6. After the reaction, a fraction of liberated lacunary anions are bound to Pb atoms at the NCs surface (as shown in Figure 5). These are indicated in eq 3 as “**1**_**b**_”, while the larger population of liberated lacunary atoms are indicated as “**1**_**f**_“, where subscripts “b” and “f” refer to “bound” and “free”, respectively.
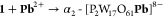
2
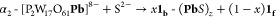
3

Finally, the Pb-coordinated anions, **1**, are more readily displaced from the PbS surface than are POMs that form electrostatically stabilized monolayers on Au NPs or POMs complexed to metal-oxide NCs via μ-oxo linkages (eq 1). This is shown by the rapid polymerization of PbS NCs upon conversion of **1** to α_2_-[P_2_V^IV^W_17_O_62_]^8**–**^ by addition of one equiv of VO^2+^ (Figure 4). This rapid metalation of bound **1** ligands is likely facilitated by labile coordination of **1** to Pb^2+^, consistent with the ^31^P NMR data in Figure 2. Indeed, lability is expected for complexation of the “soft” Lewis-acid Pb ions at the (111) surface by the “hard” Lewis-base oxo-donor ligands at the periphery of the defect site of **1**. Given this lability, coordination of **1** to PbS is likely enhanced by the large size and divalent nature of Pb^2+^.

## Conclusions

The controlled room-temperature transfer of POM-coordinated Pb^2+^ cations to S^2**–**^ anions provides ca. 4 ± 1 nm PbS NCs that, once formed, are stabilized by in situ-generated, Pb-free lacunary cluster anions. Unlike electrostatically stabilized POM protecting layers on Au NPs, or POM ligation to metal-oxide NCs via the formation of bridging-oxo linkages, α_2_-[P_2_W_17_O_61_]^10**–**^ (**1**) stabilizes PbS NCs by coordination of the formally W–O^–^ atoms at the periphery of the defect site of the cluster-anion to Pb atoms at the (111) surface of the NCs. More generally, the unique and rationally controlled metalation-induced release of stabilizing POM anions upon addition of vanadyl ions provides access to readily modifiable PbS NC interfaces^3,8,9^ for the design and fabrication of electronically conductive films^3^ for photovoltaic devices.^2,5−7^

## Experimental Section

### Materials and Instrumentation

These are provided as Supporting Information.

### Synthesis of α_2_-[P_2_W_17_O_61_]^10–^ (**1**) Stabilized PbS NCs

To a 10 mL aqueous solution of K_10_α_2_-[P_2_W_17_O_61_]·ca.10H_2_O (144.73 mg, 30.6 mmol) was added, and the solution stirred until the POM salt was fully dissolved. Then, with rapid stirring, 300 μL of Pb(NO_3_)_2_ (0.1 M, 30.0 mmol; 0.98 equiv relative to **1**) were added in two portions of 150 μL. The Pb^2+^, an aqua acid, was added in two portions to avoid a large decrease in pH that could lead to the degradation of **1**. After the rapid complexation of Pb^2+^ by **1**, the pH was ca. 5.5. Then, with rapid stirring, 1500 μL of a pH 14 solution of Na_2_S (20 mM, 30 mmol; 0.98 equiv relative to **1**) were added in 50 μL portions to avoid excursions to high pH values. During Na_2_S addition, the solution became dark brown in color; the final pH was 7.4. Characterization data are provided as the Supporting Information.

To obtain cryo-TEM images of the POM-stabilized NCs in a solution largely free of excess **1** (Figure 3B), 1 mL of EtOH was added to 2 mL of the **1**-stabilized PbS NCs prepared as described above. This resulted in precipitation of excess **1**, which was removed by centrifugation as a white pellet, while no colored material was precipitated. UV–vis spectra obtained before and after EtOH addition and centrifugation revealed an expected decrease in the 290 nm absorption band of **1**.

## Supporting Information Available

Materials and methods; histogram used to calculate average PbS NC size; phase identification of the core of **1**-stabilized PbS NCs (high-resolution TEM), and cryo-TEM images of a **1**-stabilized PbS NC solution after the addition of 0.75 equiv of VO^2+^ (PDF)
