# Supplementary material for: Polyoxometalate Ligation of PbS Nanocrystals
Source: Inorg Chem. 2025 Apr 28;64(18):8952–7. doi: 10.1021/acs.inorgchem.5c00293 (PMC12076545; doi:10.1021/acs.inorgchem.5c00293)
Supplement: Supplementary file 1 — ic5c00293_si_001.pdf [file ic5c00293_si_001.pdf]

Supporting Information

Polyoxometalate Ligation of PbS Nanocrystals

Talia Ambar[a], Aranya Kar[a], Mark Baranov[b], Nitai Leffler[a], Alevtina Neyman[a], and Ira A. Weinstock[ab]*

[a]*Dept. of Chemistry, Ben-Gurion University of the Negev, Beer Sheva, Israel*
[b]*Ilse Katz Institute for Nanoscale Science & Technology, Ben-Gurion University of the Negev, Beer Sheva, Israel*
*Email: iraw@bgu.ac.il

Contents — Page no.

Contents	Page no.
**Experimental section**	S2-S4
**Figure S1**. ^{31}P-NMR spectral documentation of Pb^{2+} transfer from α_2-$[P_2PbW_{17}O_{61}]^{8-}$ to S^{2-}.	S5
**Figure S2.** Electron diffraction of **1**-stabilized PbS NCs.	S5
**Figure S3.** XPS spectra of **1**-stabilized PbS NCs.	S6
**Figure S4.** Histogram used to calculate average PbS NC size.	S6
**Figure S5.** Phase identification of the core of **1**-stabilized PbS NCs (electron diffraction).	S7
**Figure S6.** Phase identification of the core of **1**-stabilized PbS NCs (high-resolution TEM).	S7
**Figure S7.** Intensity-percent distribution from DLS measurements of **1**-stabilized PbS NCs with different amounts of added vanadyl ion.	S8
**Figure S8.** Cryo-TEM images of a **1**-stabilized PbS NC solution after the addition of 0.75 eq. of VO^{2+}.	S8
**Figure S9**. Cryo-TEM images of a **1**-stabilized PbS NC solution after the addition of 1.0 eq. of VO^{2+}.	S9
**References**	S9

Experimental section

Materials

All materials were purchased as reagent grade and used without further purification. Lead (II) nitrate (99.999% Pb, referred to as $Pb(NO_3)_2$, Strem Chemicals, US), sodium sulfide nonahydrate (>98%, referred to as Na_2S, sigma aldrich, US), silver nitrate (>99%, referred to as $AgNO_3$, sigma Aldrich, US), copper (II) chloride dihydrate (referred to as $CuCl_2$, sigma Aldrich, US), NaCl (analytical grade, Frutarom, Israel), Vanadyl sulfate (referred to as $VOSO_4$, fluka chemicals, US), 1-butanethiol (Holland Moran, Holland), ethanol (signa Aldrich, US), toluene anhydrous (99.8%, Tzamal D-Chem, Israel), HCl (analytical grade, Bio Lab, Ltd., Israel). $K_7\alpha$-$[PW_{11}O_{39}]$,[1] $K_{10}\alpha$-$[P_2W_{17}O_{61}]$,[2] $K_9\alpha$-$[AlW_{11}O_{39}]$[3] and $K_7\alpha$-$[AlVW_{11}O_{40}]$[3] (waters of crystallization not shown) were prepared according to published procedures.

Instrumentation and methods

pH Measurements. pH values were measured using a Thermo SCIENTIFIC, ORION STAR A211 pH meter, or a EUTECH INSTRUMENTS, cyberscan pH 11 pH/ mv/ °C Meter. Prior to use, the pH meter was calibrated using standard reference solutions (pH 4.01, 7.00 and 10.01).

NMR (^{31}P). ^{31}P NMR was acquired on a Bruker 400 MHz instrument, and. Chemical-shift values were externally referenced to either 1.0 M or 85% H_3PO_4, set to $\delta = 0$ ppm. Internal lock signals were tuned using D_2O. Spectral data were processed using the NMR software package MestRecNova.

Dynamic Light Scattering (DLS). DLS data was collected at 25 °C using two instruments: 1. ALV-CGS-8F instrument (ALV-GmbH, Germany). The regular angle of measurements was 90 degrees geometry of the detector with respect to incident beam (otherwise will be noted) and the CONTIN method was used to obtain hydrodynamic radii (Rh). 2. Malvern Zetasizer Nano S90, with size measurement from 0.3 nm (diameter) to 5 microns using 90 degree scattering optics. Zetasizer software was used to obtain particle diameter. The DLS data were obtained to ensure that colloidal solutions of POM-protected metal-oxide NPs contained significant concentrations of particles in the size range of interest.

Electron Microscopy (TEM and HRTEM). Samples for dry TEM and high-resolution TEM (HRTEM) were prepared by pipetting 5-10 μL of the aqueous sample solution onto Cu grids covered with thin carbon-support films and dried in air. TEM data were obtained using a FEI Tecnai 12 G^2 electron microscope (120 kV) equipped with a Gatan slow-scan camera. HR-TEM

data were obtained using a JEOL JEM-2100F instrument operated at an accelerating voltage of 200 kV.

Cryogenic sample preparation for TEM (cryo-TEM). The cryogenically frozen samples were prepared using a fully automated vitrification device ("Vitrobot"). First, 2 μL of the sample solution were placed by pipette onto a glow discharged 300 Mesh Cu grid covered with a lacey-carbon film, held inside a 100% humidity chamber. The grid was then mechanically "blotted" and immediately plunged into liquid ethane (b.p. 185K) cooled by liquid nitrogen (b.p. 77K). Data were collected on the FEI Tecnai 12 G^2 instrument (120 kV) and the Gatan slow-scan camera, using a low-dose regime (to slow down the crystallization of vitrified water and to delay the formation of other artifacts due to beam damage). All images from both dry- and cryo-TEM (including electron diffraction patterns) were analyzed using Digital Micrograph Gatan Inc. software.

Spectroscopy (FTIR) Fourier-transform infra-red (FTIR) spectra were acquired using a Nicolet Impact 410 spectrophotometer (KBr pellets), analysed with Omnic 7 software in transmission mode.

Induction Coupled Plasma Optical Emission Spectroscopy (ICP-OES). Elemental analysis was performed using a SPECTRO ACROS ICP-OES analyser calibrated using standard solutions of the respective elements.

Synthesis

$K_{10}[P_2W_{17}O_{61}]$ (1) stabilized PbS nanocrystals. See Experimental section in the text of the article**.**

Lead sulfide. 300 μL of 0.1 M $Pb(NO_3)_2$ were mixed with 1.5 mL of 20 mM Na_2S. The product, a dark precipitate was washed with water and dried under vacuum by rotary evaporation.

Stability tests behind the results summarized in Table 1 of the text. This method is a variation on an approach used by Talapin.[4] When a solution containing α_2-$[P_2W_{17}O_{61}]^{10-}$ (**1**) stabilized PbS NCs was salted out by adding NaCl (to 1 M final concentration) followed by centrifugation, the material re-dissolved in pure water, and this was observed for up to three cycles of salting out, precipitation by centrifugation and re-dissolution. The same stability, i.e., three cycles of salting out and re-dissolution to give clear solutions, was observed for PbS NCs prepared using α-$[PW_{11}O_{39}]^{7-}$ (K^+ salt). When PbS was synthesized using K^+ salts of α-$[AlW_{12}O_{40}]^{5-}$ or α-$[AlV^{IV}W_{11}O_{40}]^{7-}$ (entries 3 and 4 of Table 1 of the

text), the PbS precipitated after one salting out with NaCl had aggregated and could only be partially re-redissolved in water, and the second cycle of salting out and centrifugation gave totally insoluble material.

An addition indication of binding by the lacunary site of **1** was obtained by using solutions of **1** to re-dissolve precipitated PbS NCs: Notably, when solution used to re-dissolve the pellets contained added **1** (3 mM) the material could be completely redissolved to give clear solutions for up to 7 precipitation / re-dissolution cycles. We note that while **1** effectively stabilizes PbS NCs, these nano-structures are much less robust than POM-stabilized metal-oxide NCs, which can undergo numerous cycles of salting out and re-dissolution with no loss of solubility or observable degradation.[5-10] The lability of **1** bound to Pb atoms at the (111) surface of PbS NCs is consistent with interaction of the "hard" Lewis base, $W\text{-}O^-$, donor groups on **1**, and the "soft" Lewis acid Pb atoms on PbS (see also Figure 2 of the text where this lability is documented by ^{31}P NMR).

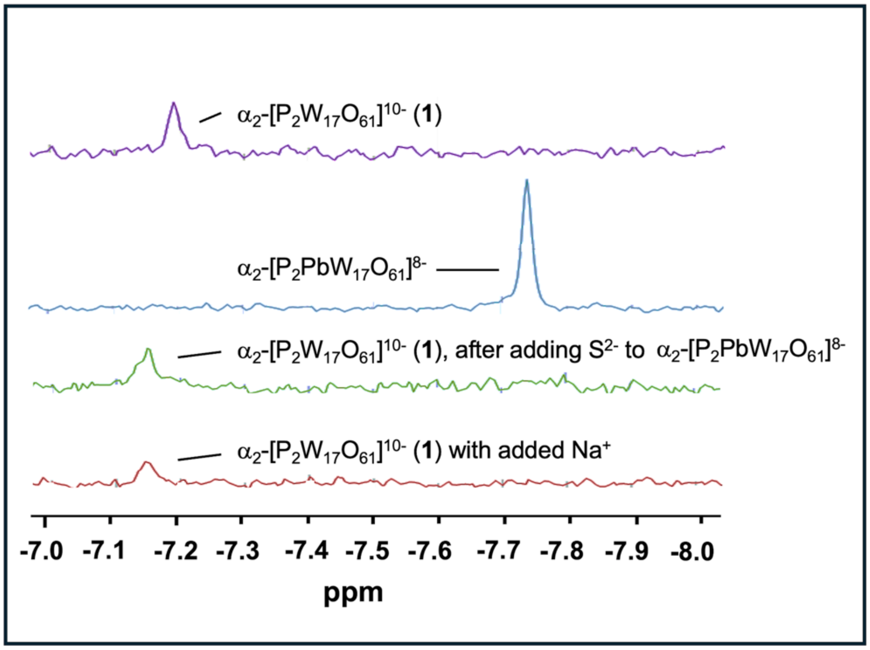

Figure S1. ^{31}P-NMR spectral documentation of Pb^{2+} transfer from α_2-$[P_2PbW_{17}O_{61}]^{8-}$ to S^{2-}. Signals arise from the P atom closest to the lacunary site. Purple (top) spectrum: Pure α_2-$[P_2W_{17}O_{61}]^{10-}$ (**1**). Blue spectrum: α_2-$[P_2PbW_{17}O_{61}]^{8-}$. Green (bottom) spectrum: Recovery of α_2-$[P_2W_{17}O_{61}]^{10-}$ (**1**), via demetalation of α_2-$[P_2PbW_{17}O_{61}]^{8-}$ upon delivery of the Pb^{2+} ion to S^{2-} to give **1**-stabilized PbS NCs. Red (botton) spectrum: α_2-$[P_2W_{17}O_{61}]^{10-}$ (**1**) in the presencer of added sodium chloride (control for identification of signal assigned to the Pb^{2+} complex, which is shifted slightly by the presence of Na^+ present from reaction with Na_2S).

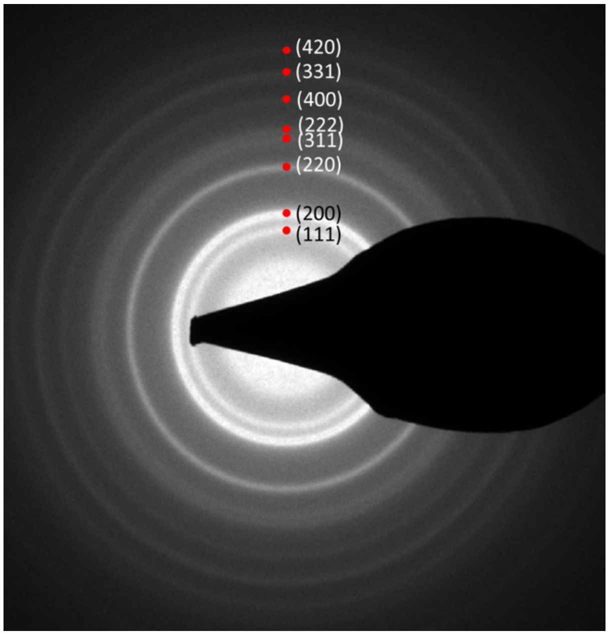

Figure S2. Electron diffraction of PbS prepared in the absence of **1**, indicative of galena-phase PbS (ICSD 63095).

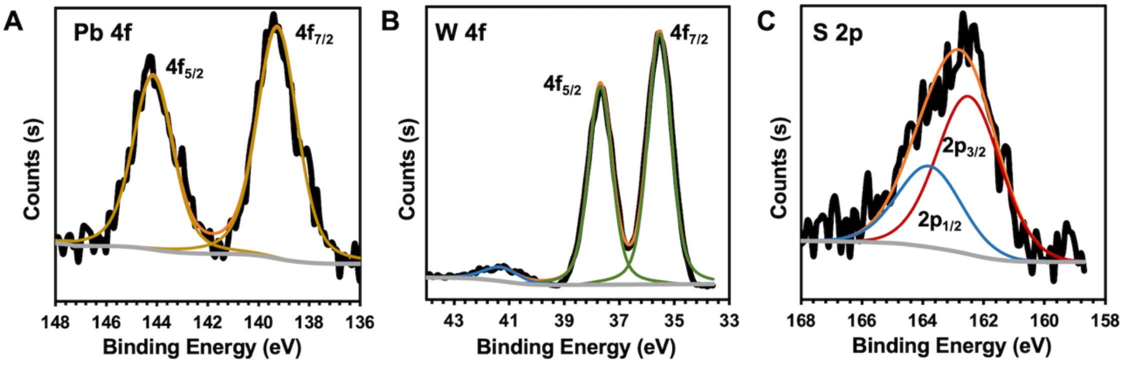

Figure S3. XPS spectra of **1**-complexed PbS. A) Pb 4f scans showing two peaks for Pb $4f_{7/2}$ (139.3 eV) and Pb $4f_{5/2}$ (144.2 eV), assigned to Pb^{2+} in PbS, B) W 4f scans showing two peaks for W $4f_{7/2}$ (35.5) and W $4f_{5/2}$ (37.7) due to presence of W^{6+} in the POM cluster, and C) S 2p scans showing two peaks for S $2p_{3/2}$ (162.3) and S $2p_{1/2}$ (163.5) due to the sulfide ions in PbS.

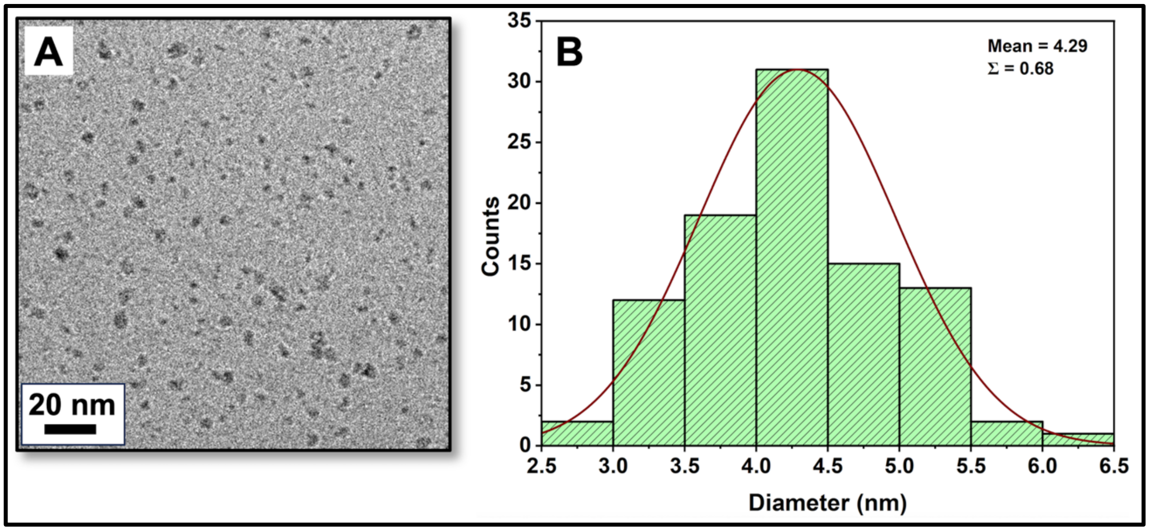

Figure S4. A) Wide area cryo-TEM image of **1**-stabilized PbS NCs measured in order to generate **B**), a histogram of NCs sizes based on the measurments of 95 particles. The distribution gave an average diameter of 4.29 nm with a standard deviation of 0.68.

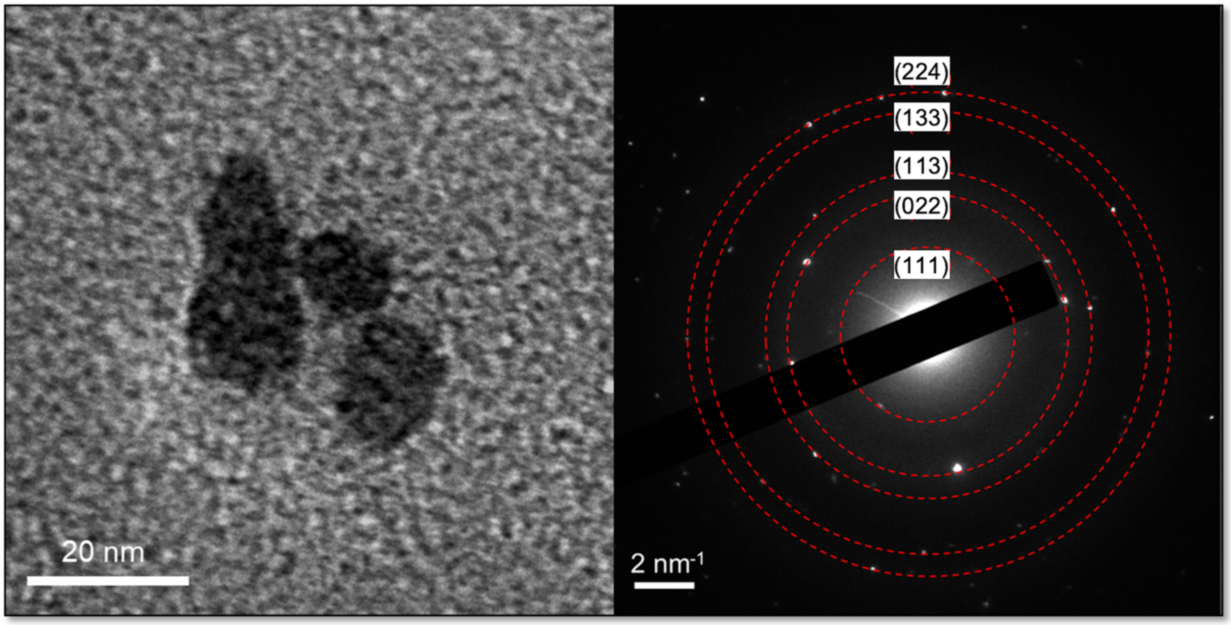

Figure S5. Phase identification of the core of **1**-stabilized PbS NCs. Left: TEM image from which a selected area electron diffraction (SAED) was obtained. Right: SAED showing the single crystalline nature of the PbS NCs, with main diffraction spots indexed, and corresponding diffraction rings are overlaid as visual guides to emphasize crystallographic orientation.

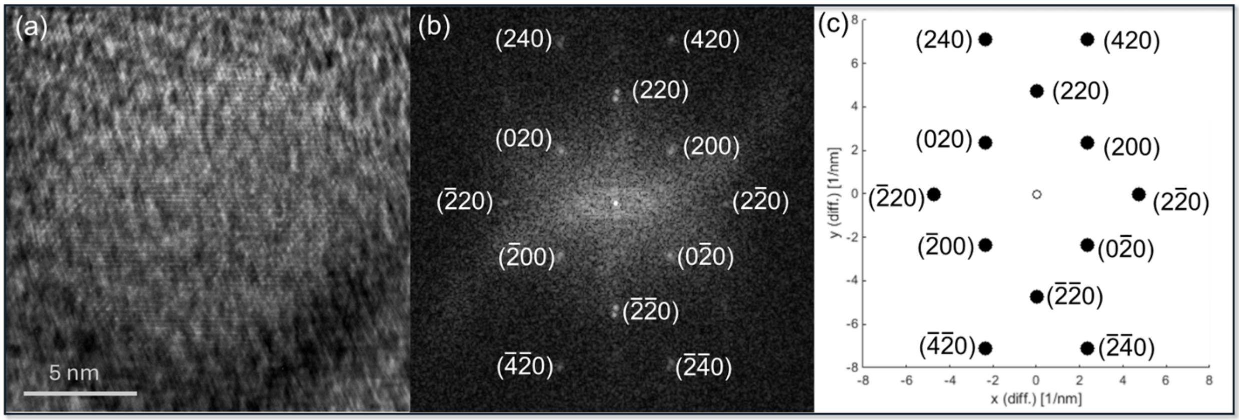

Figure S6. HRTEM analysis of POM-complexed PbS nanocrystals. (a) HRTEM image of a single PbS NC, illustrating its single-crystalline structure. (b) FFT of the HRTEM image in (a), showing indexed diffraction spots corresponding to the crystal lattice. (c) Simulated diffraction pattern along the [001] zone axis (ZA), accurately reproducing the observed diffraction spots.

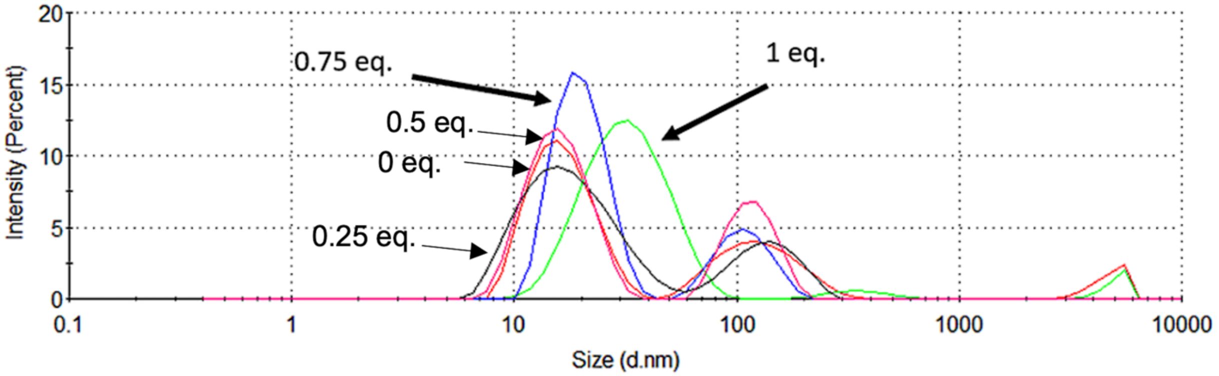

Figure S7. Intensity-percent distribution from DLS measurements of **1**-stabilized PbS NCs with different amounts of added vanadyl ion. Red: 0 eq. of VO^{2+} added. Black: 0.25 eq. of VO^{2+}. Pink: 0.5 eq. of VO^{2+}. Little change was observed immediately and 1.5 hours after adding 0.25 and 0.5 equiv. Much larger changes were observed, however, after adding 0.75 eq. of VO^{2+} (blue curve), and 1.0 equiv. (green curve).

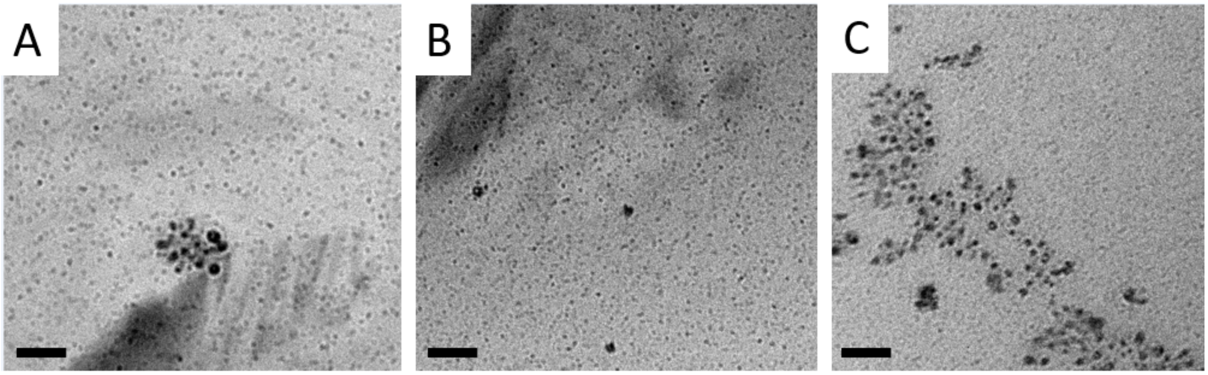

Figure S8. Cryo-TEM images of a **1**-stabilized PbS NC solution after the addition of 0.75 eq. of VO^{2+}. **A**) **A**) Small cluster of aggregated PbS NPs surrounded by free **1** and α_2-$[P_2W_{17}O_{61}VO]^{8-}$. **B**) Individual NCs of PbS possibly stabilized by remaining **1**, and with free **1** and α_2-$[P_2W_{17}O_{61}VO]^{8-}$ in the background. **C**) A large aggregate of PbS NCs partially stabilized by remaining **1**, and with free **1** and α_2-$[P_2W_{17}O_{61}VO]^{8-}$ in the background. Scale bars: 20 nm.

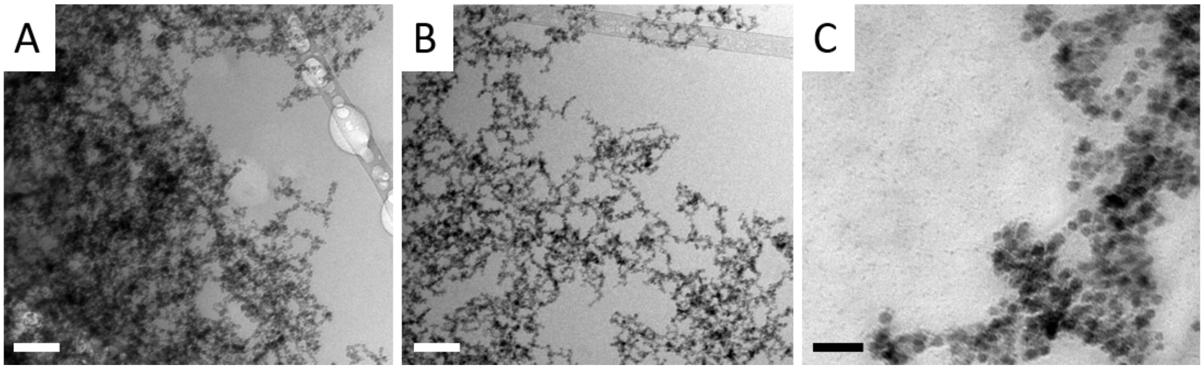

Figure S9. Cryo-TEM images of a **1**-stabilized PbS NC solution after the addition of 1.0 eq. of VO^{2+}. **A**) and **B**) Large, branched-polymer aggregates before their precipitation into insoluble material. White scalebars: 100 nm. **C**) shows a close-up of the NPs, that while aggregated, are still ca. 4 ± 1 nm in diameter, with free α_2-$[P_2W_{17}O_{61}VO]^{8-}$ in the surrounding matrix. Black scale bar: 20 nm.

REFERENCES

1. Haraguchi, N.; Okaue, Y.; Isobe, T.; Matsuda, Y., Stabilization of Tetravalent Cerium Upon Coordination of Unsaturated Heteropolytungstate Anions. *Inorg. Chem.* **1994,** *33*, 1015-1020.
2. Contant, R.; Klemperer, W. G.; Yaghi, O., Potassium Octadecatungstodiphosphates(V) and Related Lacunary Compounds. In *Inorg. Synth.*, 1990; Vol. 27; pp 104-111. https://doi.org/10.1002/9780470132586.ch18,
3. Cowan, J. J.; Bailey, A. J.; Heintz, R. A.; Do, B. T.; Hardcastle, K. I.; Hill, C. L.; Weinstock, I. A., Formation, Isomerization and Derivatization of Keggin Tungstoaluminates. *Inorg. Chem.* **2001,** *40* (26), 6666-6675.
4. Huang, J.; Liu, W.; Dolzhnikov, D. S.; Protesescu, L.; Kovalenko, M. V.; Koo, B.; Chattopadhyay, S.; Shenchenko, E. V.; Talapin, D. V., Surface functionalization of semiconductor and oxide nanocrystals with small inorganic oxoanions (PO_4^{3-}, MoO_4^{2-}) and polyoxometalate ligands. *ACS Nano* **2014,** *8*, 9388-9402.
5 Kumar Tiwari, C.; Roy, S.; Tubul-Sterin, T.; Baranov, M.; Leffler, N.; Li, M.; Yin, P.; Neyman, A.; Weinstock, I. A., Emergence of visible-light water oxidation upon hexaniobate-ligand entrapment of quantum-confined copper-oxide cores. *Angew. Chem., Int. Ed.* **2023,** *62*, e202213762.
6. Baranov, M.; Duan, Y.; Leffler, N.; Avineri, S.; Ezersky, V.; Weinstock, I. A., Entrapment of metastable nanocrystals by polyoxometalates. *Chem. Comm.* **2023,** *59*, 4364-4367.
7. Duan, Y.; Chakraborty, B.; Tiwari, C. K.; Baranov, M.; Tubul, T.; Leffler, N.; Neyman, A.; Weinstock, I. A., Solution-state catalysis of visible light-driven water oxidation by macroanion-like inorganic complexes of γ-FeOOH nanocrystals. *ACS Catal.* **2021,** *11*, 11385-11395.
8. Chakraborty, B.; Gan-Or, G.; Duan, Y.; Raula, M.; Weinstock, I. A., Visible-light-driven water oxidation with a polyoxometalate-complexed hematite core of 275 iron atoms. *Angew. Chem., Int. Ed.* **2019,** *58*, 6584-6589.
9. Chakraborty, B.; Gan-Or, G.; Raula, M.; Gadot, E.; Weinstock, I. A., Design of an inherently-stable water oxidation catalyst. *Nature Commun.* **2018,** *9*, 4896.
10. Raula, M.; Gan Or, G.; Saganovich, M.; Zeiri, O.; Wang, Y.; Chierotti, M. R.; Gobetto, R.; Weinstock, I. A., Polyoxometalate complexes of anatase-titanium dioxide cores in water. *Angew. Chem., Int. Ed.* **2015,** *54*, 12416-12421.
